# Non-Hodgkin lymphomas and ionizing radiation: case report and review of the literature

**DOI:** 10.1007/s00277-021-04729-z

**Published:** 2021-12-08

**Authors:** Inge Schmitz-Feuerhake, Rainer Frentzel-Beyme, Roland Wolff

**Affiliations:** 1 University of Bremen, Bibliotheksstraße 1, 28359 Bremen, Germany; 2 Society for Radiation Protection, Grenzstraße 20, 30627 Hannover, Germany

**Keywords:** Chronic lymphocytic leukaemia, Target organ, Lymph nodes, Occupational disease, X-ray diagnostics

## Abstract

Non-Hodgkin lymphoma (NHL) increased continuously since the last century in developed countries. While they are considered as disease in elder ages, a remarkable increasing incidence is also observed in German children and juveniles. The higher rates are interpreted by the changes in classification because diseases such as chronic lymphocytic leukaemia were also identified as NHL. Considerable rates of NHL were found in nuclear workers and liquidators of Chernobyl, i.e. in cases of low-dose chronical exposures. In Germany, we noticed three workers who developed NHL after decontamination of nuclear facilities. The bone marrow is generally considered as target organ for ionizing radiation, but NHL is obviously induced in the whole pool of lymphocytes. Therefore, the dosimetry in cases of typical occupational external and internal exposure must be revised. A high radiation sensitivity for NHL is a possible suspect and likely reason which may partly explain the continuous rise of the diseases in populations underlying the current increases of medical diagnostic exposure. NHL is also induced in children and juveniles with a history of diagnostic X-rays.

## Introduction

Because many cell types are involved in the immune reaction of the circulating cellular system which may undergo malignant transformation, there are also many types of non-Hodgkin lymphoma (NHL). They were re-classified by IARC of the WHO [1]. The changes are of high complexity [2]; they are therefore assumed to be the explanation of the observed continuous increase of the incidences in industrial countries like Germany [3].

In former times, it was believed by experts that radiation-induced NHL could be expected only after high doses as in cohorts of the Japanese A-bomb survivors, after radiation therapy, or in patients exposed to thorium dioxide used as contrast medium [4]. Systematic registration of NHL in workers began in 1950 when the US Public Health Service initiated a program in order to control the health effects occurring in the uranium industry. Archer and co-workers [5] studied the cancer mortality among uranium mill workers and detected remarkable excess deaths due to malignant diseases of the lymphatic and hematopoietic tissue other than leukaemia, predominantly lymphomas. They explained this result by already established findings about the accumulation of uranium and thorium in the tracheobronchial lymph nodes in animals.

Observations of excess incident cases of NHL in occupationally exposed persons became more frequent in the last decades and led often to surprisingly high relative risk rates, among them large cohorts of so-called liquidators. These are survivors of about 800,000 men who were deployed by the Soviet Union for shielding and decontamination tasks after the explosion of the Chernobyl reactor in 1986.

Chronic lymphocytic leukaemia (CLL) was formerly considered as non-radiogenic, based on a lack of findings in the Japanese A-bomb survivors. This dogma was criticized by Richardson and co-workers in 2005 [6]. Later on, CLL became classified as a subgroup of NHL and categorized as an indolent type of NHL [1].

We noticed three cases suffering from illness of B-lymphocyte proliferation (2 CLL and one B-cell lymphoma) who were involved in the decontamination of closed nuclear establishments, since Germany began to dismantle the installations of nuclear energy. The cluster of cases gave reason to evaluate the upcoming knowledge about radiation-induced NHL.

## Observations in Germany

The men we refer to had worked in several nuclear enterprises, and also in a facility producing nuclear fuel rods including mixed oxide fuel assemblies (MOX) containing enriched uranium and plutonium. Their working periods and diagnoses are shown in Table 1.

**Table 1:** CLL and NHL in workers A–C after decontamination of MOX facility

Client	Diagnosis	Year of first diagnosis	Diagnosis at age	Period of occupational exposure	Working with MOX	External dose mSv
A	Indolent B-cell NHL	2012	46y	1989–2011	2001–2006	116
B	B-CLL	2012	49y	1996–2013	1996–2000	108
C	B-CLL stage B by Binet	2014	52y	2002–2005	2002–2005	46

According to biomonitoring data, they were exposed to penetrating gamma radiation of 116, 108 and 46 mSv (Sv is the unit representing the so-called effective dose; the dose limit for occupationally exposed workers is 0.1 Sv=100 mSv in 5 years.) Any dose contribution by incorporated radionuclides was estimated to be very low by the Governmental Association of Occupational Insurances (Berufsgenossenschaft für Rohstoffe und chemische Industrie).

Between 1996 and 2006, the 3 patients had been deployed by their same employer to the same former facility for fuel assemblies before their jobs in the decontamination. The employer documented that the places for decontamination had contained MOX fuel. This was certainly done in order to point to the special risks of plutonium, which delivers high organ doses in the case of incorporation. Indeed, we derived from some sporadic measurements by whole body counting, in urine and from the presence of neutrons, that there must have occurred incorporation of uranium and plutonium, probably by inhalation of radioactive dust. The indices for a remarkable internal dose contribution were discussed in a former paper [7].

The clustering of the diseases which are normally very rare in ages up to 52 points towards an origin by occupational exposure.

## Epidemiological findings about non-Hodgkin lymphomas in A-bomb survivors

German regulations for recognition and compensation of radiation-induced occupational diseases still regard NHL and CLL as different entities and they are assumed to be originated in tissues of low radiation sensitivity [8]. This evaluation refers to recommendations of the committee UNSCEAR^1^ in 2006 [9] who chose the survivors of the atomic bomb explosions at Hiroshima and Nagasaki in 1945 as their main reference collective for health effects by radiation in humans.

Significant excess of NHL in the Japanese A-bomb survivors was not reported before 1994, and only in men [10]. This may be due to the fact that these diseases were extremely rare in the Japanese population, and that the latency periods after exposure are normally very long [6, 11]. More recent results of the Life Span Study (LSS) in Hiroshima and of the cohort “Not in city” are listed in Table 2.

**Table 2:** Non-Hodgkin lymphomas in the Life Span Study (LSS) and the “Not in city” cohort of the Japanese A-bomb survivors

Collective	Number of exposed persons	Mean follow-up y	Cases observed *n*	Relative risk* RR	Doubling dose Sv
I. LSS Analysis 2013 Incident cases NHL [12]	45,000	31.9	402	1.1	2.2 (men only)
II. LSS Analysis 2009 Mortality NHL [11]	20,940	28.2	73		0.236 (men only)
III. Not in city 1977 Incident cases CLL [13]	26,508		28	1.5	
IV. LSS Analysis 2013 Incident cases CLL [12]	45,000	31.9	12	Increase signif.	

*The relative risk RR is the number of observed cases divided by the expected cases

The LSS is the usual reference and is related to the direct penetrating radiations by gamma rays and neutrons. A possible contribution by radioactive fallout or residual radiation by neutron activation in the grounds or other materials is assumed to be neglectable. The update of the incidence in 2013 (Study I in Table 1) leads to a doubling dose of 2.2 Sv^2^ [12]. This is in contrast to a former analysis of Richardson and co-workers of 2009 [11] using the same database. They regarded the mortality in men above the age of 15 years at exposure only in the dose range below 500 mSv and considered the fact that the deaths occurred predominantly more than 35 years after exposure. Their lowest result about a doubling dose of 236 mSv was calculated assuming a 10-year lag between exposure and death (Study II in Table 2).

The long-lasting dogma about CLL as being non-radiogenic ignored the fact that the Japanese cohort “Not in city” had shown an excess of CLL (III in Table 2). These people had not been in the cities at time of the bombing and were not exposed by the direct radiation during the explosion. They entered some days later in order to seek for relatives or to help the victims. They were formerly assumed to be a suitable control population. They were, however, exposed by fallout which deposited predominantly outside the cities, and by residual radiation near the hypocentres. Later studies of the effects by fallout in the air and residual exposures published in 2008 showed remarkable cancer risks [13].

Excess cases of CLL in the “Life Span Study” were not registered before 2001 (IV in Table 2). This resulted in a significant dose-effect relationship which is interpreted as proof for causation by radiation [12]. A risk figure was not derived.

The very low incidence of NHL including CLL in Japanese people requires excluding this cohort as a reference for European people; the significant difference in incidence may be determined by genetic differences. Another point is the type of exposure, which was a flash of high dose rate delivered from the bomb. This results in other conditions of cell distortion and repair as in cases of low dose rate chronical exposure at workplaces. The catastrophic situation which leads to the “survival of the fittest” has been shown to contribute to an underestimation of radiation risks in normal populations [14, 15].

## Dosimetry and target organ for radiation-induced NHL

In the pertinent literature, radiation-induced CLL and NHL are usually related to the exposure of the bone marrow. However, B-cell lymphomas which are the dominant type of observed malignant diseases of the lymphatic system are originated in mature lymphocytes [1]. These predominantly circulate outside the bone marrow and will be the target for somatic mutation by ionizing radiation. As mentioned in 1973 by Archer et al. [5], an accumulation of radionuclides occurs in the lymph nodes which is explained by the immunological reaction of macrophages functioning as scavengers of particles such as alpha emitters. Enhanced concentration of nuclear fuel isotopes of uranium, thorium and plutonium after incorporation was found in autopsy studies [16–20].

It is, therefore, justified to assume that this enhancement effect has led to the occurrence of the described 3 lymphomas in German workers who inhaled plutonium, because the effect is hard to explain by the measured external exposure (Table 1).

The bone marrow dose equals the mean dose, if there is only external irradiation and homogenous dose distribution in the body as is assumed in the case of the atomic bomb survivors. If only parts of the body are exposed as for medical diagnostics or in cases of incorporation of radionuclides, the dose to the peripherical lymphocytes will remain unknown.

This conclusion is confirmed by a study in 3440 patients in whom 12 NHL occurred after local radiotherapy for cancer at the nasopharynx [21]. The investigation was carried out after nasopharyngeal radiation application of radium. Because of the limited area of exposure, the mean bone marrow dose was estimated to be only 5 mSv. This led to a value ERR/Sv = 280 and a doubling dose for the effect of only 4 mSv which cannot be regarded as a reliable result or estimate.

## NHL in occupationally exposed men

Findings about non-Hodgkin lymphomas in occupationally exposed persons are compiled in Table 3. For historical reasons, they are also divided into NHL and CLL based on the terminology used in the original publications. Women are rarely employed in most of the relevant professions and were excluded.

**Table 3:** Epidemiological studies on radiation-induced non-Hodgkin lymphomas in men (*RR* relative risk, *n.e.* not estimated, *n.s.* not significant)

No. Disease	Study	Study population	Study type	Number of exposed persons	Mean follow-up y	Number of cases	Dose Sv mean or range	Results		Remarks
								RR	Doubling dose Sv	
1 NHL	USA 1973 [5]	Workers in Uranium mills	Mortality	715	2–13	4	n.e.	3.9		
2 NHL	USA 2001 [22]	Nuclear technique, weapons Atomics Int. + Rocketdyne workers 1955–1993	Mortality	4563 2207		28 8	≥0.2 ≥0.03	15.7 44.6		External exposure Internal exposure
3 NHL	USA 2009 [11]	Atomic weapons Savannah River workers 1950–2002	Mortality	15,264	34.4	51	0.0–0.5		0.131	10-y lag
4 NHL	USSR 1994 [23]	Mayak workers 1958–1986	Mortality	1812	34	25	0.02–0.7	3.5		High-dose group ICD 200-208
5 NHL	USA 1984 [24]	Radiologists 1920–1939 1940–1969	Mortality	8500			n.e.	2.73 0.41		
6 NHL	U.K. 2001 [25]	Radiologists 1897–1979	Mortality	2698	25.8	9	n.e.	3.1		
7 NHL	USA 2016 [26]	Radiologists entrance <1940	Mortality	1426		19	n.e.	2.69		
8 NHL	Japan 1999 [27]	Rad. technologists Entrance 1897–1933 Entrance 1934–1950	Mortality	4595 7600	22.4 22.3	15 2	n.e.	1.48 n.s. 0.56		
9 NHL*	USA 2008 [28]	Nuclear shipyard workers	Mortality	28,000	12.8	50	n.e.	5.8		≥ 10 mSv; 5-y lag *including other lymphopoietic cancer
10 NHL	USA 2012 [29]	Flight attendants	Mortality	1701	31	10	Median 0.013	2.30		
11 NHL	U.K. 1999 [30]	Sellafield reprocessing nuclear facility Workers 1971–1986	Incidence ICD 200-209	11,635	28.6	15	0.164	3.8	0.059	20-y lag Including multiple myelomas
12 NHL	U.K. 2021 [31]	Nat. Registry Rad. Workers 1950–2011	Incidence	172,452	22.9	711	0.025	1.03	0.90	Including 10% women 10-y lag***
13 NHL	Internat. 2005 [32]	Metaanalysis flight attendants	Incidence	10,551	< 19.3	6	n.e.	2.49		
14 CLL	USA 2007 [33]	Workers in 5 nuclear facilities	Mortality	94,500		43	Median 0.011	1.36 n.s.	0.05	Case-control study < 100 mSv
15 NHL CLL	Internat. 2008 [34]	Liquidators Belarus, Russia, Baltic states	Incidence	146,000		20 21	Median 0.013**		0.036** 0.213**	Case-control study
16 CLL	Ukraine 2016 [35]	Liquidators 1987–2012	Incidence	152,520		80	n.e.	3.6		Cases until 2006
17 CLL	Czech Repl. 2006 [36]	Uranium miners	Incidence	23,043		42		2.0		
18 CLL	Germany 2010 [37]	Uranium miners	Incidence	360,000		159		circ 2		Case-control study
19 CLL	USA 2010 [38]	Uranium mines, U& Radium refining, processing Workers 1932–1978	Incidence	16,770	22	22			0.137***	

**Bone marrow dose

***See text

No. 1 in Table 3 represents the above-mentioned historical work of Archer and co-workers [5]. The study is based on mortality data as many of the other investigations (Nos. 2–10). These studies contain the information that NHL is induced below the legal dose limits. In order to gain risk figures for radiation induction, the problem must be seen that indolent lymphomas have a good prognosis for survival under therapy which improved considerably in the last decades. In 2015–2016, the German incidence rate for NHL (ICD-10 C82-C88) in men was 16.9 while the mortality was only 5.4, which means that about 66% of the diseased cases would not be registered in a mortality study [3].

This unavoidable underestimation of effects in mortality studies will certainly lead to erroneous judgements about absent radiation risks in low-dose occupations such as in a study still published in 2020 based on a huge cohort of 110, 297 radiologic technologists who were, however, exposed to only 8.5 mGy^3^ of mean dose and followed up not longer than 22 years on average [39].

Studies based on incidences would, therefore, be preferable in order to derive risk estimates. Among these, in Table 3 (No. 12), one result gathered in 2016 from the British National Registry for Radiation Workers (NRRW) with 711 of NHL cases in a very large cohort [31] missed CLL by not being included because the authors still regard them to be a separate disease [40]. Assuming dose-effect proportionality, they derive a value for NHL of ERR per Sv of 1.11 (0.02–2.69) which leads to a doubling dose of 0.90 Sv which is in some contradiction to the other findings in Table 3. The results in the updated NRRW study are gained, however, by assuming a 10-year lag between the time of exposure and the date of diagnosis while the mean follow-up was only 22.9 years. The possible loss of cases in the first 10 years of observation, which is not reported by the authors, may have led to an erroneous result.

There are 3 other incidence studies regarding NHL in former classification (Nos. 11, 13, 15). Study No. 11 in workers of the British reprocessing plant Sellafield combines, however, lymphomas and multiple myelomas [30]. It found the highest relative risk of 3.8 assuming a 20-year lag. The estimated mean effective dose of 164 mSv is gained assuming the highest solubility class for inhaled Pu. The incidence of NHL was 2-fold higher in workers with incorporated Pu in comparison to other radiation workers. This study shows a high radiation sensitivity of the lymphatic system but the specific exposure conditions may not be suited to derive quantitative risk estimates for NHL representative for other cohorts.

The increase in flight personnel (No. 13) shown by SIR=2.49 (1.03–6.05) is surprising considering the rather low official estimates of their exposure and the short follow-up of <19.3 years [32]. It corresponds to the NHL effect in the mortality study No. 10 in flight attendants resulting in SMR=2.30 (1.10–4.23). Because of the lacking dose estimation, a figure for radiation risk cannot be derived from this study.

The incidence study in liquidators (No. 15) refers to a combined collective of men from Belarus, Russia and Baltic countries [34]. The estimated doses are derived for the bone marrow which may be the reason for the resulting very low doubling dose of 36 mSv for NHL. This risk figure is therefore also not suitable as a reference.

CLL were found to be the most frequent form of leukaemia in liquidators [41]. This is confirmed in studies Nos. 15–16.

The exposure of uranium miners as well as of workers in processing facilities for uranium and radium by radon and uranium/radium dust is probably not really to quantify because of the problems with accumulation in the lymph nodes after incorporation (Nos. 17–19).

## Radiation-induced NHL in children and juvenile persons

Lymphomas as a causally related effect were found in former [42] and recent studies about childhood cancer after X-ray diagnostics. Rajaraman et al. [43] studied the possible sequels in a case-control study based on 2690 childhood cancer cases of UK born between 1976 and 1996. Excluded were investigations by CT because of their higher exposures compared to other X-ray radiographs. Exposure in early infancy (0–100 days) resulted in a “small” non-significant excess risk for all cancers and an increased risk of lymphoma with an odds ratio of 5.14 (1.27 to 20.8) based on 7 observed cases, 6 of them classified as NHL (OR=6.85).

Cancer cases after exposure in utero (*n*=120) resulted in non-significant increase; for NHL (*n*=13), the odds ratio was 1.48 (0.66 to 3.32). Dose estimates were not given. The authors suggest cautious use of diagnostic imaging procedures to the abdomen/pelvis of the mother during pregnancy and in children at very young ages.

Mathews et al. [44] investigated cancer risks in 680,000 Australian people exposed to CT scans in childhood or adolescence, aged 0–19 years on January 1985 or born between 1985 and 2005. Overall cancer incidence was 24% higher for exposed than for unexposed persons. Lymphoid cancers were also named as relevant being significantly increased. NHL and multiple myeloma were not separated; based on 65 cases, they showed a relative risk of 1.70 (1.31 to 2.20).

The authors state that the observed increase of cancer was mostly due to irradiation. They emphasize the fact that the life time risk of the exposure could not be estimated in their investigation because the mean duration of follow-up after exposure was only 9.5 years. It is also to consider in the study of Rajaraman et al. [43] (see above) that they included only cancers in persons below the age of 15 and therefore probably miss the majority of induced lymphomas because of their longer latencies.

Lymphomas were also found in a German study in children after CT scans [45]. The authors regarded cancer cases between 1980 and 2010 from the German Childhood Cancer Registry (GCCR) and according CTs from 20 hospitals. 63,214 CT investigations were found for 39,184 cases. A significantly increased incidence was found for all cancers and for lymphomas. 10 lymphomas were observed while 3.4 were expected which led to SIR=2.96 (1.42–5.45). Part of the cancers were, however, classified as correlated to patients of “high risk”, i.e. these were assumed to have a disposition associated with a higher cancer risk. 5 of the lymphomas were therefore excluded, one of the patients suffering from cancer at time of the first CT and the other 4 cases with a post-transplant lymphoproliferative disorder. This resulted in non-significant SIR=1.54 (0.50–3.59) for lymphomas. A significant increase of all cancers remained (SIR=1.49). It must again be considered that all cancers occurred in childhood and therefore the lifetime risk of the exposure was not investigated. The mean follow-up between exposure and cancer diagnosis was only 4.1 years [46].

A continuous increase of NHL in Germany is also seen in children. According to the GCCR, the incidence rose from 1986 to 2016 by about 35% [47].

Leukaemia in childhood as a consequence of X-ray exposure in utero is an accepted effect by UNSCEAR and the International Commission on Radiological Protection ICRP. It was detected in the 1950s in the Oxford Survey of Childhood Cancers (OSCC). Other cancers occurring after birth were also studied.

Bithell and Stewart [48] refer on 719 lymphoma cases in their case-control study based on 8519 deaths from cancer in childhood (<15 years) observed in the period 1953–1967 after in utero exposure. They derived a relative risk for radiation-induced lymphomas of 1.4 (1.1–1.7), which was related to X-ray exposures between 1938 and 1967. A “Committee Adrian” was installed in 1957 by the British Ministry of Health in order to attain reliable dose estimates. They regarded two diagnostic procedures which led to an exposure in utero: obstetrical abdominal X-rays, which were intended to image the entire foetus; and pelvimetric X-rays, which were used to show the bony structure of the maternal pelvis and part of the foetus within to provide information to facilitate delivery. Their best estimates for both techniques, applied in most cases, resulted in foetal doses of 5.8 mSv and 13 mSv [49].

## Results and conclusions

NHL can certainly be induced by low doses of ionizing radiation received by the lymphatic tissues which are of high relative radiation sensitivity. Precise radiation risks are not available, however, even in cases of complete homogenous dose distribution because of the varying classification of the diseases over time. If only parts of the body are exposed, any dose estimation will be unsafe because the target lymphocytes are distributed all over the circulatory system in varying concentrations. Especially in cases of incorporation of radioactivity by macrophages, any dose estimation for NHL will become close to impossible.

The survey of data from low-dose exposures (Table 3) and our own experience leads to the conclusion that NHL must be expected predominantly at work places where inhalation of alpha emitting nuclides is not avoidable: in uranium mines and processing of nuclear fuel materials, in nuclear facilities and nowadays at places where such facilities are demolished. The enhancing effect by the accumulation of nuclides in the lymph nodes leads to exposures of the lymphocytes which are not determinable from outside and thus unquantified. Hence, the total dose of the critical organ will be systematically underestimated under typical radiological exposure situations. Radiation-induced NHL should therefore be considered as a special problem of protection for radiation workers and have to be adequately assessed in compensation cases.

From the data in Table 3, the doubling dose for adult men and chronical exposure can be assumed to lie below 200 mSv—in contrast to the result in the British worker study [30]. The continuous increase of NHL in populations of the last decades may partly be explained by the continuous increase of X-ray diagnostics by CT.

## Data Availability

All dosimetric and medical data which are published by the authors were provided from the patients.

## Footnotes

1 United Nations Scientific Committee on Atomic Radiation

2 The doubling dose is the organ dose which induces the same incidence in the exposed cohort as is observed in the reference population. The relative risk is then RR=2.

3 1 mGy corresponds to 1 mSv in exposures by X-rays and gamma rays

## Code availability

There is no code.
